# Improved STD Syndrome Management by a Network of Clinicians and Pharmacy Workers in Peru: The PREVEN Network

**DOI:** 10.1371/journal.pone.0047750

**Published:** 2012-10-17

**Authors:** Patricia J. García, Cesar P. Carcamo, Geoff P. Garnett, Pablo E. Campos, King K. Holmes

**Affiliations:** 1 Epidemiology, STI/AIDS Unit, School of Public Health, Universidad Peruana Cayetano Heredia, Lima, Peru; 2 Imperial College, London, United Kingdom; 3 Department of Epidemiology, University of Washington, Seattle, Washington, United States of America; 4 Department of Medicine, University of Washington, Seattle, Washington, United States of America; 5 Department of Global Health, University of Washington, Seattle, Washington, United States of America; 6 Center for AIDS & STD, University of Washington, Seattle, Washington, United States of America; Vanderbilt University, United States of America

## Abstract

**Background:**

Sexually Transmitted diseases (STD) syndrome management has been one cornerstone of STD treatment. Persons with STD symptoms in many countries, especially those with limited resources, often initially seek care in pharmacies. The objective of the study was to develop and evaluate an integrated network of physicians, midwives and pharmacy workers trained in STD syndromic management (The PREVEN Network) as part of a national urban community-randomized trial of sexually transmitted infection prevention in Peru.

**Methods and Findings:**

After a comprehensive census of physicians, midwives, and pharmacies in ten intervention and ten control cities, we introduced seminars and workshops for pharmacy workers, and continuing education for physicians and midwives in intervention cities and invited graduates to join the PREVEN Network. “Prevention Salespersons” visited pharmacies, boticas and clinicians regularly for educational support and collection of information on numbers of cases of STD syndromes seen at pharmacies and by clinicians in intervention cities. Simulated patients evaluated outcomes of training of pharmacy workers with respect to adequate STD syndrome management, recommendations for condom use and for treatment of partners. In intervention cities we trained, certified, and incorporated into the PREVEN Network the workers at 623 (80.6%) of 773 pharmacies and 701 (69.6%) of 1007 physicians and midwives in private practice. Extremely high clinician and pharmacy worker turnover, 13.4% and 44% respectively in the first year, dictated continued training of new pharmacy workers and clinicians. By the end of the intervention the Network included 792 pharmacies and 597 clinicians. Pharmacies reported more cases of STDs than did clinicians. Evaluations by simulated patients showed significant and substantial improvements in the management of STD syndromes at pharmacies in intervention cities but not in control cities.

**Conclusions:**

Training pharmacy workers linked to a referral network of clinicians proved feasible and acceptable. High turn-over was challenging but over come.

## Introduction

Sexually transmitted diseases (STD) syndrome management has been one cornerstone of STD treatment.[1] In developing and developed countries, antimicrobial self-medication is common.[2] Persons with STD symptoms in many countries, especially those with limited resources, often initially seek care in pharmacies.[3], [4] Pharmacies have long service hours, offer prompt access, free advice, and are known and trusted by communities served.[5] For reproductive health and STD, pharmacies play a particularly important role. In Africa,[6]–[8] South and Central America,[9]–[11] East Asia,[12]–[14] and elsewhere, pharmacies often represent the first point of contact with the health system for many persons with STD symptoms. In the US, pharmacies have become involved in expedited therapy, prior to or without clinical examination, for persons exposed to certain sexually transmitted infections (STIs). In the UK, community pharmacists who have completed accredited training can provide antibiotics.[15], [16] Access to antimicrobials via the internet is also increasing rapidly.[17].

Thus public health strategies for STI control cannot ignore the role of pharmacies. One approach, motivated in part by concerns about over-use of antimicrobials and emerging antimicrobial resistance, involves enforcement of regulations proscribing antimicrobial dispensing by pharmacies without prescription. Efforts to enforce such regulation significantly decreased antimicrobial consumption in Chile,[18] but have been variably or inconsistently effective in other settings.[19] Another approach involved training of pharmacy workers to improve antimicrobial prescribing practices for STD syndrome management in Nepal,[20] Vietnam,[21] Ecuador,[22] and Peru.[23].

In Peru we have evaluated incremental enhancements of training programs for pharmacy workers linked to training of clinicians, designed to strengthen management of four STD syndromes: urethral discharge (UD), vaginal discharge (VD), genital ulcer disease (GUD), and pelvic inflammatory disease (PID). Initially, we assessed the impact of short didactic training on management of these four syndromes, using simulated patients to evaluate impact.[10] Next, we conducted a district-randomized trial of a more intensive training program in Lima: a series of seminars and workshops were followed by ongoing structural support from “prevention salespersons,” linked to simultaneous training of physicians. Pharmacy workers were trained to recognize and directly manage UD and VD; and to recognize and refer GUD and PID to trained and certified clinicians. Again simulated patients documented a large impact of training. [23].

To formally test the impact of such a program on the population-level prevalence of the STIs associated with these four syndromes, as part of a multicomponent urban community-randomized trial of STI prevention in Peru[24], we created the PREVEN Network of physicians, midwives, and pharmacy workers, all trained in STD syndromic management, as one of four key intervention modalities. This paper describes the training program for the Network, rates of attrition from the Network over the three year intervention period, numbers of cases of STD syndromes reported by the Network over three years, and evaluation by simulated patients of STD syndrome management at pharmacies in ten intervention and ten control cities during the trial.

## Methods

### Ethical Statement

Institutional Review Boards at the University of Washington (Seattle USA), Universidad Peruana Cayetano Heredia (UPCH), and US-NMRCD (the US U.S. Naval Medical Research Center Detachment in Lima Peru) approved the protocol and instruments. All IRBs exempted pharmacy workers and clinicians from informed consent.

Twenty cities with populations between 50,000 and 300,000 located on the coast, the Andean region or the jungle of Peru were randomized in pairwise fashion into intervention and control arms. The ten intervention cities received a multicomponent intervention. One of the four intervention modalities involved training of clinicians and pharmacy workers in improved recognition and management of STD syndromes, including STI/HIV prevention counseling, resulting in formation of the PREVEN Network. The intervention began in July 2003 and continued through December 2006.

### Training of Pharmacy Workers, Physicians and Midwives

Potential Network members were pharmacy workers, physicians, and midwives working in private practice and at public health settings. Leaders representing each group in intervention cities, including physicians, midwives, pharmacists, and pharmacy workers were recruited to help develop the strategy for improving STD syndromic management. These leaders helped form discussion panels involving representatives of regional governments, Network leaders, and other civil society members, to discuss management of STD syndromes and creating sustainable strategies for prevention and control of STIs including HIV infection in the city.

A comprehensive map-based, street-by-street census of pharmacies and boticas (drugstores not owned or managed by pharmacists, but allowed to sell all pharmaceuticals, henceforth also referred to as pharmacies), and of physicians and midwives [25], [26] was conducted in each of the 20 intervention and control cities in early 2003. We updated the censuses throughout the study (updated for clinicians only in intervention cities). In the ten intervention cities, training interventions targeted all pharmacies and all private practice physicians and midwives who agreed to participate. Strategies previously developed, evaluated, reported [23] and based on well described strategies like academic detailing [27] included: (1) seminars and workshops for pharmacy workers; (2) use of “Prevention Salespersons” for ongoing structural support of the network; and (3) continuing education for physicians and midwifes.

Pharmacy seminars and workshops included four 90-minute training sessions with small groups of eight to ten pharmacy workers who met over lunch with two training specialists (a pharmacist and a midwife). Interactive workshops included case-oriented discussions, role-playing, and self-evaluation, with certification upon successful completion. Training emphasized recognition of STD syndromes, syndromic management guidelines, and referral of clients with GUD or PID to the PREVEN Network of certified clinicians. The final session provided training in client-centered counseling by pharmacy workers using motivational interviewing [28] to promote treatment of sex partners, and future condom use for casual and commercial sex. Trainees learned to assess the client’s actual and perceived risk, reduce barriers to risk-reduction, and support behavior change and promote partner treatment. Training materials are available at www.proyectopreven.org.

### “Prevention Salespersons”

“Prevention salespersons” were pharmacists and midwives who received 50 hours of specialized training on STDs, health education, and counseling and communication skills, and were responsible not only for conducting the prevention seminars, but also for providing follow-up structural support to certified Network members, making monthly visits to each pharmacy and clinician in the Network, distributing educational materials for pharmacy workers and for pharmacy clients, and answering questions and discussing concerns. Pharmacy workers themselves suggested the “Prevention Salesperson” approach–analogous to that of pharmaceutical salespersons–as an innovation to provide ongoing peer-mediated diffusion of information.

Because of frequent pharmacy and botica worker turnover, the “Prevention Salesperson” provided continued on-site training for new pharmacy workers, with the participation of any initially trained and certified pharmacy workers who remained on site.

### Continuing Education for Clinicians

Continuing education (CE) is not mandatory in Peru for physicians or any health professional. We decided to introduce a CE program for physicians and midwifes in private practice -about half of whom also worked in the public sector- based on a training curriculum which took into account the adult learning process, [29] self-taught learning, and distance education. It consisted of: a) an initial four-hour induction workshop; followed by b) one month of individual learning guided by a self-instruction manual and access to a training support system through the PREVEN webpage, telephone calls, and visits from the local team; and c) a final consolidation workshop. Based on our prior training program which included 1,100 physicians in Lima,[23] this training emphasized guidelines for STD syndromic management, patient counseling on condom use, and partner treatment. Following completion of training, clinicians were evaluated, certified, and invited to join in the PREVEN Network. A directory of trained and certified clinicians was posted on the website and distributed to all members of the network in the ten intervention cities. “Prevention Salespersons” also made monthly visits to the physicians and midwives of the Network, and distributed CE credit materials. Additionally, from August 2005 through March 2006, a substantial interactive Internet-based STD management course for physicians (reported separately) was carried out in the ten intervention cities.[30].

### Health Communication Campaign

Also, we developed a 2003–05 health communication campaign targeting young adults to enable them to recognize STD symptoms, and to promote early health care-seeking for such symptoms, utilizing the PREVEN Network. We implemented this health communication campaign locally in each intervention city using pamphlets, posters, local media, radios and local TV.

### Evaluation

Process and output measures included percentages of pharmacies, pharmacy workers, physicians, and midwives completing training and continuing in follow up; numbers of activities undertaken and promotional materials distributed by “Prevention Salespersons”; and monthly reports of STD syndromes seen by Network pharmacies and clinicians in intervention cities.

“Prevention Salespersons” paid monthly visits in intervention cities to each pharmacy, physician, and midwife in the PREVEN Network, asking them to estimate numbers of clients with UD, VD, GUD, or PID seen during the last week. These numbers were extrapolated to four weeks to estimate numbers seen per month. Results are presented as STD cases reported by Network members.

We employed and trained simulated patients to present clinical vignettes (scripted scenarios), to evaluate certified pharmacy workers management of UD, VD, and GUD, and their recommendations for condom use and for partner treatment. For each evaluation, we randomly chose 30 pharmacies in each of the ten intervention and control cities, and compared management of the three syndromes through visits by different simulated patients for each syndrome. No simulated patient visited any pharmacy more than once. For UD, management was considered adequate if the patient was offered treatments for gonorrhoea and chlamydial infection that conformed to national guidelines (ciprofloxacin 500 mg orally plus azythromycin 1 g orally) or was referred to a PREVEN Network clinician. We allowed simulated patients to buy medications for up to $10 US when necessary to avoid the “artificiality” of the interaction between the simulated patient and the pharmacy worker and so as not to curtail recommendations from pharmacy workers for partner treatment or condom use. For abnormal VD, management was considered adequate if the patient was offered treatment with 2 g single oral dose of metronidazole or was referred to a PREVEN Network clinician. For symptoms of GUD, management was considered adequate only if the patient was referred to a PREVEN Network clinician. Evaluations by simulated patients were done at baseline and at three, six, and 18 months post-training, for each of the three syndromes, using previously described methodology. [10].

We also performed sentinel surveillance of STD cases seen at pharmacies from control and intervention cities at three, nine, and 18 months post-initiation of the intervention. These results are reported separately.

### Data Management and Statistical Analysis

All information was collected in standardized formats, by local teams in each city. Data were reviewed for consistency and entered into a data base for analysis. For the simulated patient analysis we compared intervention and control cities with unconditional logistic regression by using cities as clusters using generalized estimating equations (GEE) to control for intracluster correlation. Statistical analyses were performed using STATA 8·0 for Windows (Stata Corporation, Texas, USA).

## Results

### Numbers and Percentages of Pharmacy Workers, Physicians and Midwives Trained and Certified

In the initial 2003 census of the ten intervention cities we identified 773 pharmacies and boticas, 2,292 pharmacy workers, 629 physicians, and 378 midwives. All were invited to participate in the program. Pharmacy workers were organized in groups according to their past training and role at the pharmacy: 23·6% were pharmacists (with university degree), 36·7% were pharmacy technicians (1–3 years technical training), 11% were nurse technicians, 18·7% were pharmacy owners with no specific training in pharmacy, and 10% were relatives of owners or others with no specific training. Most (62%) were women; median age was 34·6 years for men (range 13 to 86) and 31·8 years for women (range 15 to 75). Those 18 years or older were invited to participate in training. Characteristics of private physician and midwife populations have been described elsewhere.[25], [26].

After training, pharmacy workers averaged 18 of 20, and physicians and midwives 19 of 20, in correct responses to questions on STD syndromic management, with improvement in correct responses from pre-training to post-training tests of 54·8% for pharmacy workers and 40% for physicians and midwives. Most pharmacy workers and most physicians and midwives in private practice in each city were trained, certified and recruited to the PREVEN Network (Table 1).

**Table 1:** Baseline census and training of pharmacy/botica workers, physicians and midwives in private practice in the 10 intervention cities.

Pharmacies and boticas (P&B)	
Number of P&B according to 2003 census	773
Number of pharmacy and botica workers according to 2003 census	2292
Number of P&B participating in training (Aug 2003–Nov 2003)	696 (90%)
Number of standardized seminars given (average 8–10 workers/seminar)	686
Percent knowledge improvement	54·8%
Number of P&B certified*	642 (83%)
Number of pharmacy and botica workers certified	2074 (90·5%)
Number of P&B initially affiliated with the PREVEN Network	623 (80·6%)
**Physicians and midwives in private practice (P&M)**	
Number of P&M according to 2003 census	1007
Number of P&M participating in training (Aug 2003–Nov 2003)	810 (80·4%)
Number of workshops (20–30 clinicians in each)	51
Percent knowledge improvement	40%
Number of P&M certified**	728 (72·3%)
Number of P&M initially affiliated with the PREVEN Network	701 (70%)

* Certification required attendance at all four seminars and passing the peer evaluation test and at least 60% correct answers to the written test.

** Certification required attendance at two seminars, completion of homework and at least 60% correct answers to the written test.

### Follow Up

During the trial, as Network participants left their jobs or moved to other cities, new pharmacy workers, physicians, and midwives were trained, certified, and added to the Network. Of the pharmacies initially enrolled, 119 (19%) closed during the first year and 183 (29·3%) closed during the three year trial period, including 39 (19·6%) of the true pharmacies and 144 (34%) of the boticas, while 352 new pharmacies opened including 74 true pharmacies and 278 boticas. The rate of turnover of pharmacy workers was exceptionally high. During the first year alone, 920 (44%) of the pharmacy workers left their jobs, and during the three years of the trial 1,677 (80·8%) of our initial trainees left, including 457 (76·2%) leaving a true pharmacy and 1,220 (82·8%) leaving boticas. However, we managed to train and certify 1,928 new pharmacy workers during this period. We also experienced substantial loss of physicians and midwives from the Network, including 94 (13·4%) in the first year and 286 (41%) during the three year period, while 182 new clinicians joined the Network during this period (Table 2). Rates of turnover of physicians and midwives were nearly identical.

**Table 2:** Follow up of PREVEN Network members in 10 intervention cities, (2003–2006).

Pharmacies and boticas (P&B)	N (%)*
Number of P&B initially affiliated with the PREVEN Network	623
Number of P&B closed during the intervention period 2003–2006	183 (29·4%)
Number of new P&B included in the PREVEN Network during 2004–2006	352 (56·5%)
Number of P&B trained and active at the end of the intervention	792 (127·1%)
**Pharmacy and botica workers (PBW)**	
Number of PBW trained in the PREVEN network in 2003	2074
Number of PBW lost to follow up during the intervention period 2003–2006	1677 (80·9%)
Number of new PBW trained and included in PREVEN network during 2004–2006	1928 (93·0%)
Number of PBW trained and active at the end of the intervention	2325 (112·1%)
**Physicians and midwives in private practice (P&M)**	
Number of P&M in PREVEN network in 2003	701
Number of P&M lost to follow up during the intervention period 2003–2006	286 (40·8%)
Number of new P&M trained and included in PREVEN network during 2004–2006	182 (26%)
Number of P&M active at the end of the intervention	597 (85·2%)

* Percent in relation to 2003 PREVEN Network participants from each category.

More than 250,000 educational pamphlets regarding STIs and condoms were distributed to clients of pharmacy workers and clinicians. Additionally more than 50,000 posters, calendars, stickers, manuals, and other pieces of merchandising related to the PREVEN Network were distributed during the intervention.

### STD Cases Reported by Members of the PREVEN Network

**Figure 1:**
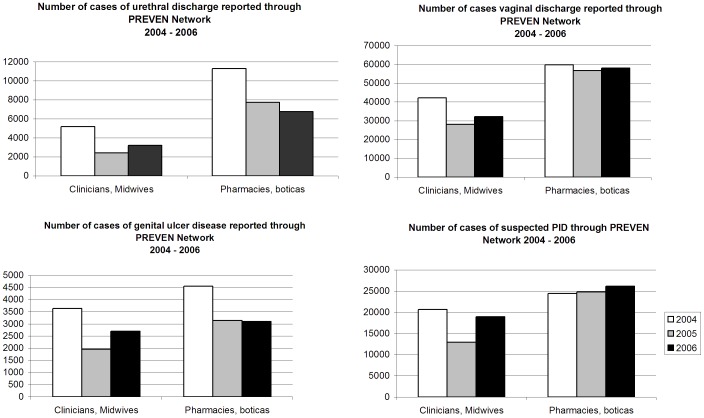
Number of cases of STDs reported by PREVEN Network. The number of cases of urethral discharge, vaginal discharge, genital ulcer disease, and pelvic inflammatory disease reported by the PREVEN Network of pharmacies or boticas for 2004, 2005, and 2006 was substantially higher than the numbers reported by Network physicians and midwives, especially for urethral discharge, less so for suspected PID.

In the ten intervention cities, the number of cases of STD syndromes reported to the “Prevention salesperson” from Network pharmacies was substantially higher than the numbers reported by Network physicians and midwives for 2004, 2005 and 2006, especially for urethral discharge, less so for suspected PID (Figure 1). Numbers of cases varied across cities (not shown). No such data were collected from control cities.

### Evaluation by Simulated Patients

**Figure 2:**
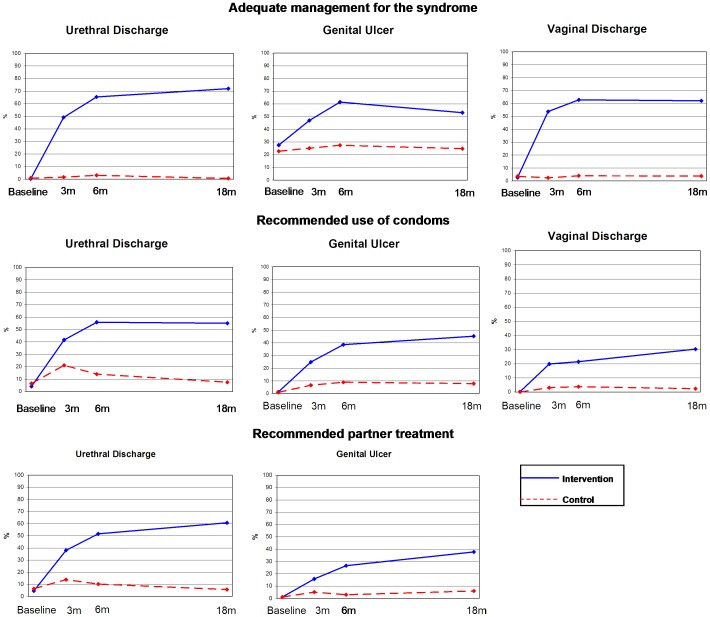
Results of evaluations by Simulated Patients. Evaluations to pharmacy workers at baseline both at intervention and control cities showed no significant differences in STD management or referral, or in recommendations for use of condoms or partner treatment. Subsequent evaluations at three, six and 18 months showed significantly better performance for all measures in intervention cities.

A total of 7,280 simulated patient visits were made to the 30 pharmacies randomly chosen in each of the ten intervention and ten control cities during the trial. At baseline, intervention and control cities showed no significant differences in syndromic therapeutic management or referral, or in recommendations for use of condoms or partner treatment, but subsequent evaluations at three, six and 18 months showed significantly better performance for all measures of management in intervention cities. Substantial improvement in practice was sustained for 18 months following completion of initial training (Figure 2).

## Discussion

Training pharmacy workers in STD syndrome management and creating a linked referral network of trained and certified clinicians proved feasible and acceptable at the community level. We emphasized referral of GUD and suspected PID to a clinician, because of the need for serologic testing for syphilis, and the complexity of the treatment including injection of benzathine penicillin in GUD management, and the need for physical examination and potentially for laboratory testing in women with symptoms of PID. In a recent paper, Khan [31] found poor performance of druggists, even after training, in the management of GUD and PID.

Efforts to implement similar programs may similarly require continuous training to deal with high worker and facility turn-over. Turnover of boticas was far more frequent than for pharmacies, both for closing and for opening of new boticas. Regulations for boticas are less rigorous, but they provide a very large percent of the market for dispensing pharmaceuticals in Peru, representing almost 70% of the baseline census of pharmacies and boticas and 80% at the end of the intervention. Workers in actual pharmacies themselves represented a heterogeneous population with only 60% having received formal pharmacy training. Attention at the policy level to academic and occupational professionalism seems important to reduce the turn-over of pharmacy and botica workers. The rate of turn-over of physicians and midwives was approximately half that of pharmacy workers. Follow up of all physicians and midwives lost to the Network, suggested most if not all actually left the intervention cities. Migration of health professionals within the country and especially emigration to other countries is well known but not well documented in Peru. The National Institute of Statistics and Informatics [32] reports that the annual number of physicians who have traveled outside the country and not returned within 6 months increased from 420 in 2000 to 1,543 in 2007. Discussions regarding brain drain are active in Peru, [33] but even mobility within the country presents a challenge for achieving training, for keeping trained people where they are needed, and especially for creating and sustaining a health network system. However, the online version of the course developed for clinicians [30] is now used by the College of Professional Midwives in Peru to offer training on STIs and represents an innovative approach to continuing education.

Evaluations by simulated patients strongly supports the effectiveness of STD syndromic management training of pharmacy workers. Simulated patients were blinded to the intervention and we changed the teams for each evaluation. We cannot rule out an influence of the presence of posters and STD merchandising information on the evaluations in intervention cities, but our systematic post-evaluation interviews of simulated patients provided no evidence of such bias. Improvement in pharmacy workers performance over time is perhaps attributable to reinforcement by the visits of “Prevention Salespersons” and to more “practice” and confidence acquired by the workers.

Although the number of physicians and midwives certified (728) in 2003 exceeded the number of pharmacies certified (642), substantially more cases of UD and VD in particular were seen by pharmacies than by the clinicians in the Network. One limitation is that we do not have a way of validating the number of clients with UD, VD, GUD or PID reported seen at pharmacies.

Strengths of this study include the lessons learned from previous extensive studies with pharmacies and clinicians in Lima that guided design of this intervention modality for the multicomponent intervention trial. The baseline census of pharmacies and clinicians prior to the intervention enabled us to plan activities and define intervention targets.

We believe that the simultaneous training of pharmacy workers and clinicians and linking trained pharmacies workers to the trained physicians and midwives in a Network, not only encouraged cross-referrals including referral of genital ulcer disease and suspected PID from pharmacies to clinicians, but enhanced the acceptability of the Network to pharmacy, physicians’, and midwives’ sectors. The creation of the “Prevention salesperson” support mechanism was also useful and could be used for other social and public health benefits. The impact of the multicomponent intervention at the population level is reported separately.

We conclude that opportunities exist to train and empower pharmacy workers, physicians, and midwives in the management of STD syndromes, creating a network that provides better quality in the management of STIs. Training was feasible, well accepted at the community level and appears effective in terms of process and outcome indicators.
